# Zero- to low-field relaxometry of chemical and biological fluids

**DOI:** 10.1038/s42004-023-00965-8

**Published:** 2023-08-04

**Authors:** Seyma Alcicek, Piotr Put, Adam Kubrak, Fatih Celal Alcicek, Danila Barskiy, Stefan Gloeggler, Jakub Dybas, Szymon Pustelny

**Affiliations:** 1 Goethe University Frankfurt, University Hospital, Institute of Neuroradiology, 60528 Frankfurt am Main, Germany; 2 Institute of Physics Faculty of Physics, Astronomy and Applied Computer Science, Jagiellonian University in Kraków, 30-348 Kraków, Poland; 3 Faculty of Chemistry, Jagiellonian University in Kraków, 30-387 Krakow, Poland; 4 Jagiellonian Center for Experimental Therapeutics, Jagiellonian University in Kraków, 30-348 Kraków, Poland; 5 Helmholtz Institute Mainz, GSI Helmholtz Center for Heavy Ion Research GmbH, 55128 Mainz, Germany; 6 Institute of Physics, Johannes Gutenberg-Universität, 55128 Mainz, Germany; 7 Max Planck Institute for Multidisciplinary Sciences, 37077 Göttingen, Germany

**Keywords:** Biophysical chemistry, Solution-state NMR, Sensors, Biophysical chemistry, Blood proteins

## Abstract

Nuclear magnetic resonance (NMR) relaxometry is an analytical method that provides information about molecular environments, even for NMR “silent” molecules (spin-0), by analyzing the properties of NMR signals versus the magnitude of the longitudinal field. Conventionally, this technique is performed at fields much higher than Earth’s magnetic field, but our work focuses on NMR relaxometry at zero and ultra-low magnetic fields (ZULFs). Operating under such conditions allows us to investigate slow (bio)chemical processes occurring on a timescale from milliseconds to seconds, which coincide with spin evolution. ZULFs also minimize *T*_2_ line broadening in heterogeneous samples resulting from magnetic susceptibility. Here, we use ZULF NMR relaxometry to analyze (bio)chemical compounds containing ^1^H-^13^C, ^1^H-^15^N, and ^1^H-^31^P spin pairs. We also detected high-quality ULF NMR spectra of human whole-blood at 0.8 μT, despite a shortening of spin relaxation by blood proteomes (e.g., hemoglobin). Information on proton relaxation times of blood, a potential early biomarker of inflammation, can be acquired in under a minute using inexpensive, portable/small-size NMR spectrometers based on atomic magnetometers.

## Abstract (web summary)

NMR relaxometry can provide information on paramagnetic as well as diamagnetic compounds in solution by analyzing nuclear-spin relaxation times, and, operated at ultra-low magnetic fields, slow processes can be studied. Here, the authors employ zero and ultra-low magnetic fields using an atomic magnetometer as a simple, portable, robust, inexpensive, and sensitive tool to analyze chemical solutions and biofluids.

## Introduction

Nuclear magnetic resonance (NMR) relaxometry is a method that allows one to characterize the physical and dynamic properties of samples by analyzing nuclear-spin relaxation^1,2^. Measurements of the longitudinal relaxation time *T*_1_, governing the restoration of thermal equilibrium in a sample (corresponding to the growth of static magnetization in high-field NMR), and the transverse relaxation time *T*_2_ (determining the decay of oscillating transverse magnetization) provide valuable information about sample environment. In contrast to NMR spectroscopy, relaxometry can be performed with cheaper lower-field magnets as generally there is no need to obtain a well-resolved NMR spectrum^3^. This approach has been used in agricultural^4^, petrochemical^5^, and food^6^ sciences, as well as in the analysis of biological samples^7^.

In addition to conventional (high-field) NMR relaxometry studies, NMR relaxometry at (ultra-)low magnetic fields (ULFs) has been recently demonstrated^8–10^. This became possible due to the application of non-inductive sensors, which are sensitive to low-frequency magnetic signals (particularly in the sub-kilohertz range)^11,12^. Specifically, atomic magnetometers—offering high near-DC sensitivity, low price, small size, and non-cryogenic operation—have recently been used for such measurements^13,14^. In ULFs, proton Larmor frequencies are in the range where they overlap with the rate of many slow biochemical processes of interest that occur on the microsecond to millisecond timescale^15,16^ such as protein folding^17,18^, ligand binding^19^, membrane transport^20^, intramolecular diffusion^21^, oxidation-reduction reactions^22,23^, and chemical exchange in biomolecules^24^. Therefore, compared to conventional NMR relaxometry, operation at ULFs enables the study of slow processes, as the spin evolution in this field regime occurs at similar timescales. Furthermore, the study of NMR samples in this field regime is especially interesting, as the relaxation properties strongly depend on the magnetic-field strength. This allows for the direct determination of molecular motion parameters^14^.

NMR relaxometry can provide information on paramagnetic as well as diamagnetic (e.g., proteins) compounds in solution^10,25^. Blood is one of the attractive biological specimens for relaxometry studies. By probing the *T*_2_ relaxation of water protons, NMR relaxometry of blood samples has been shown to hold great promise in the diagnosis and prognosis of metabolic disorders (insulin resistance, dyslipidemia), infections (candidiasis, malaria) and hemostatic disorders^3,26,27^. Relaxation of water protons in blood can provide valuable information about blood proteomes as water protons form hydrogen bonds with proteins, lipoproteins, and metabolites, affecting spin relaxation. While the contribution of macromolecules (e.g., proteins and lipoproteins) to proton relaxation is considerable, the influence of small metabolites (e.g., glucose and amino acids) on the process can be neglected due to the fast molecular motion, leading to inefficient dipolar relaxation^3,28^. This may allow us to observe inflammatory-induced protein profile variations, independent of minor metabolites whose concentration may vary in an unrelated manner. Although variation in the molecular weight profile of blood proteins also affects the relaxation rate, in general, a linear correlation between protein concentration and relaxation rate is expected due to slower rotational and diffusion mobility of macromolecules^29^. Consequently, the existence of a strong correlation between proton relaxation times and protein content makes *T*_1_ and *T*_2_ values promising biomarkers^30^.

In this paper, the investigation of NMR relaxometry of chemical and biological samples in zero and (ultra-)low magnetic fields (ZULFs) is presented. First, the effect of dissolved paramagnetic oxygen on relaxation times is examined in strongly coupled heteronuclear systems at zero field. This is achieved by the analysis of zero-field NMR *J*-spectra^31^, where peaks’ positions are determined by the ^1^H-^31^P and ^1^H-^13^C *J*-couplings. Second, the influence of solvents (H_2_O and D_2_O) on relaxation process is studied in ULFs by monitoring the longitudinal relaxation of protons coupled to long-lived ^15^N in methylpyridinium. Third, NMR spectra of water solutions with different concentrations (0.2-1 mM) of CuSO_4_, which are particularly interesting for quantitative imaging studies using phantoms (to mimic biological samples)^32^, are used to study the influence of paramagnetic agents on water-proton relaxation under ULFs (10–510 μT). Finally, nuclear magnetic relaxation dispersion (NMRD) profiles of protons in human whole-blood and plasma are investigated at ULFs to demonstrate the potential of the ZULF NMR relaxometry method for the analysis of biological samples.

## Results and discussion

### Effect of oxygen on zero-field NMR

Oxygen molecules, being paramagnetic (i.e., containing unpaired electrons), influence nuclear-spin relaxation^33^. Therefore, to avoid oxygen-induced relaxation, liquid NMR samples are often degassed. This is achieved by purging the samples with neutral gas (e.g., nitrogen) or by the freeze-pump-thaw technique and subsequent flame sealing. Despite the fact that this procedure is routinely used, its significance on ZULF NMR spectra has never been investigated. To fill this gap, we studied this effect by analyzing the amplitudes and widths of peaks observed in zero-field NMR *J*-spectra^14,34^, which provide information about the longitudinal (*T*_1_) and transverse (*T*_2_) relaxation times.

**Fig. 1:**
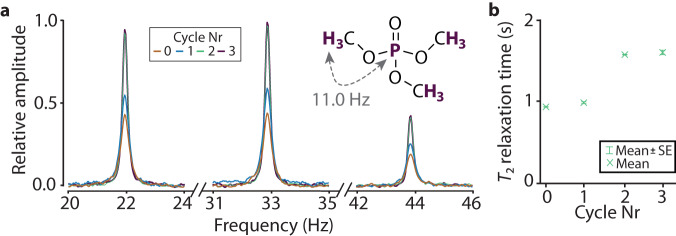
Effect of oxygen on zero-filed NMR *J*-spectra of trimethyl phosphate. **a** Zero-field NMR *J*-spectra (showing resonances at 2*J*_HP_, 3*J*_HP_, and 4*J*_HP_) of the trimethyl phosphate samples prepared with a different number of freeze-pump-thaw cycles (0, 1, 2, and 3). Each spectrum is a result of averaging 64 transients. **b** Extracted transverse relaxation time *T*_2_ of trimethyl phosphate in zero magnetic field versus the number of freeze-pump-thaw cycles performed during the sample preparation. Each experimental point and each error bar indicate the mean and standard error values of relaxation time extracted from three peaks shown in **a**, respectively.

First, we examined trimethyl phosphate solutions prepared with a procedure that involves a different number of freeze-pump-thaw cycles. As trimethyl phosphate consists of nine equivalent protons coupled to phosphorus (with coupling constant *J*_HP_ = 11 Hz), its zero-field spectrum consists of peaks at *J*_HP_, 2*J*_HP_, 3*J*_HP_, 4*J*_HP_, and 5*J*_HP_ (see detailed discussion of trimethyl phosphate spectrum at zero field in ref. ^34^). Here, due to the relatively low amplitude of the peaks at 11 and 55 Hz, we only analyzed the peaks at 22, 33, and 44 Hz. As demonstrated in Fig. 1a, an increase in the amplitude of the peaks and a decrease in their linewidth (full-width at half maximum, FWHM: 0.34(6) Hz, 0.32(8) Hz, 0.20(3) Hz, and 0.19(9) Hz after 0, 1, 2, and 3 cycles, respectively) were observed up to two freeze-pump-thaw cycles (1 versus 2 cycles: *p* = 0.00001). Although cycling initially helps to reduce the peak linewidth, the results also show that no significant differences in the signal parameters were observed by increasing the number of cycles above two (2 versus 3 cycles: *p* = 0.48) (Fig. 1b). Changes in peak linewidths and therefore molecule relaxation time *T*_2_ are associated with the reduction of paramagnetic oxygen in the sample. Most likely, after two cycles, the oxygen level in the solution is small (potentially negligible) in terms of its contribution to relaxation. Therefore, no improvement in the signal was achieved after the second cycle. Alternatively, even though the vacuum pump can reduce pressure over the frozen liquid to about 10^−6^ mbar, it might be insufficient to remove remaining dissolved oxygen any further after two cycles.

As the next step, we investigated the influence of paramagnetic oxygen on heteronuclear long-lived spin singlet states, which are protected from intramolecular dipolar relaxation between the two spins^35^. In high-field NMR, this state has been demonstrated for homonuclear spin-1/2 pairs (e.g., parahydrogen), where two-coupled spins are magnetically equivalent, and hence can constitute a spin-0 (singlet) and a spin-1 (triplet) systems. The triplet states are symmetric, and the singlet state is anti-symmetric with respect to particle interchange. Since the main relaxation mechanism, originating from the dipolar coupling, is invariant upon exchange of two spins, it is unlikely to cause a singlet-triplet breaking-symmetry transition; thus, the dipole-dipole coupling cannot contribute to relaxation of the singlet state^35^. At a zero magnetic field, coupled heteronuclear spin-1/2 pairs can form singlet states as well^36^. Specifically, zero-field heteronuclear singlet states have been previously reported in ^13^C-formic acid and ^13^C_1_-benzene with tens of seconds of lifetimes^36^, which are significantly longer than the longitudinal relaxation times *T*_1_.

^13^C-formic acid is one of the standard samples for ZULF NMR due to its long relaxation time and simple molecular structure. The zero-field spectrum of ^13^C-formic acid consists of a single peak at about 222.2 Hz, which is the strength of the one-bond ^1^H-^13^C coupling, while the hydroxyl-group proton coupling is negligible due to a fast proton exchange. As discussed in detail in ref. ^36^, after thermal prepolarization with the permanent magnet, the sample is transferred to the ULF region, where it is stored in the guiding (storage) field for a given time. Next, the field is suddenly switched off ( ≈ 10 μs) to provide a non-adiabatic transfer to the zero field where singlet-triplet spin system occurs and hence generate coherences between the $\left\vert {{{{{{{{\rm{T}}}}}}}}}_{0}\right\rangle$ (total quantum number *F* = 1, magnetic quantum number *M*_*F*_ = 0) and $\left\vert {{{{{{{{\rm{S}}}}}}}}}_{0}\right\rangle$ (*F* = 0, *M*_*F*_ = 0) states (see ref. ^37^ for a detailed explanation). As described above, the measurements of the width of the ZULF NMR resonances provide access to the transverse relaxation time *T*_2_, while the dependence of peak amplitudes on the storage time gives information about the longitudinal relaxation time *T*_1_. In the considered case, the longitudinal relaxation is determined by the so-called slow and fast relaxation, characterized by the relaxation times *T*_*s*_ and *T*_*f*_, respectively^36^. The fast decay is caused by the equalization of the population between three triplet magnetic sublevels ($\left\vert {{{{{{{{\rm{T}}}}}}}}}_{-1}\right\rangle$ (*F* = 1, *M*_*F*_ = − 1), $\left\vert {{{{{{{{\rm{T}}}}}}}}}_{0}\right\rangle$, and $\left\vert {{{{{{{{\rm{T}}}}}}}}}_{+1}\right\rangle$ (*F* = 1, *M*_*F*_ = + 1), and the slow relaxation is associated with thermalization among the singlet and triplet states. Thereby, the bi-exponential fit to the amplitude data provides information about both relaxation times. Alternatively, relaxation may be investigated by sudden switching off of the leading field and successive application of the pulse of a magnetic field oriented along the *y*-axis while the magnetometer is sensitive along the *z*-axis (see details in the Methods section). In this case, the amplitude of the signal is proportional to the population difference between the $\left\vert {{{{{{{{\rm{T}}}}}}}}}_{+1}\right\rangle$ and $\left\vert {{{{{{{{\rm{T}}}}}}}}}_{-1}\right\rangle$ states^38^. In turn, signal decay is only determined by the lifetime of the triplet states, and hence the relaxation time *T*_1_ can be determined from a single exponent fitting to the amplitude decay (see Methods).

**Fig. 2:**
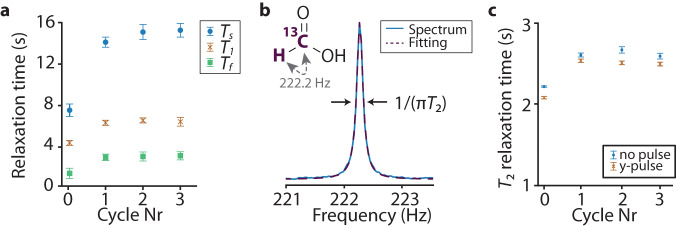
Effect of oxygen on relaxation times *T*_1_, *T*_*f*_ (fast relaxation), *T*_*s*_ (short relaxation), and *T*_2_ of ^13^C-formic acid in zero field. **a** Relaxation times *T*_1_, *T*_*f*_, and *T*_*s*_ (see the text for explanation) of ^13^C-formic acid as a function of the number of freeze-pump-thaw cycles performed during the sample preparation. **b** Lorentzian fit (dashed line) of the zero-field NMR signal of ^13^C-formic acid (solid blue line) at 222.2 Hz. **c** Comparison between the transverse relaxation times measured after a non-adiabatic transfer to zero field with (orange crosses) and without (blue circles) a complimentary *y*-pulse. Error bars indicate standard-deviation errors of the fitting parameters.

To study the influence of the amount of dissolved paramagnetic oxygen on the relaxation process mentioned above, we measured the zero-field NMR spectra of ^13^C-formic acid solutions following different numbers of freeze-pump-thaw cycles (Fig. 2a). The *T*_2_ values extracted (as demonstrated with the example spectrum in Fig. 2b) for the schemes described above (i.e., with non-adiabatic switching off the field and with the transverse *y*-pulse) are shown in Fig. 2c. An increase in all relaxation times (to a greater extent in the case of *T*_*s*_) is observed when a single and two freeze-pump-thaw cycles are performed. This is due to the efficient removal of oxygen from the samples. However, there is no significant effect of the third cycle on relaxation terms as in the case of trimethyl phosphate. It should be noted that the longest relaxation time we measured is shorter than the ones reported in ref. ^36^. The reason is that even though there is no signal associated with the proton of the hydroxyl group, the contribution of this exchangeable proton to the polarization relaxation is non-negligible^39^. In fact, increasing the proton-exchange rate enables elimination of this relaxation mechanism. However, in our study, we used neat ^13^C-formic acid (95% in weight in H_2_O), in contrast to the previous study where water and acetonitrile were mixed to accelerate the chemical exchange process. A further increase in the exchange rate leads to the decoupling of the hydroxyl-group proton from the ^13^CH_3_ group, prolonging the relaxation time and affecting the zero-field spectra, as demonstrated in refs. ^39,40^. It should be also noted that the experiments with a non-adiabatic transfer result in narrower zero-field peaks compared to the measurements with an additional transverse pulse, which is an indicator of a longer *T*_2_ value. This finding could be explained by the generation of coherences between the $\left\vert {{{{{{{{\rm{T}}}}}}}}}_{0}\right\rangle$ and long-lived $\left\vert {{{{{{{{\rm{S}}}}}}}}}_{0}\right\rangle$ states after non-adiabatic transfer, as mentioned above. On the other hand, the application of transverse pulse increases the intensity of the signal^34^.

To summarize, it is demonstrated that the presence of paramagnetic oxygen molecules has a crucial impact on ZULF NMR spectra and it should be taken into account in quantitative ULF relaxometry experiments. This finding also suggests the potential use of ZULF NMR relaxometry in the evaluation of paramagnetic gas impurities^41,42^.

### Solvent effect in ULF NMR Relaxometry

Relaxation measurements can also be performed under low-field conditions, i.e., in the regime where the Zeeman interaction begins to dominate over the scalar couplings^43,44^. This is illustrated in the ultra-low-field spectra of ^15^N-methylpyridinium (^15^N-MP) obtained under a magnetic field of 1.88 μT, which is precursor of pyridinium-derived compounds. Due to the long (several minutes) lifetime of ^15^N nuclei, biocompatibility, and the ability to be hyperpolarized in water, these compounds have been proposed as magnetic-resonance-imaging contrast agents^45^. We investigated ^15^N-MP in both H_2_O and D_2_O solutions, which allowed us to study the role of a solvent on longitudinal relaxation at ULF conditions.

**Fig. 3:**
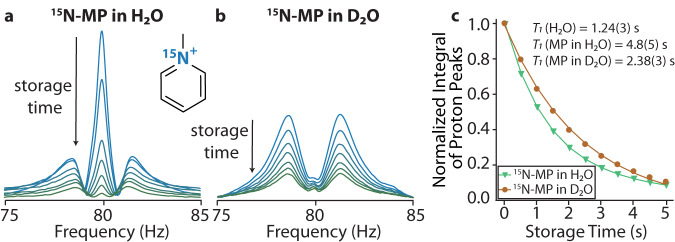
Solvent effect in ULF NMR of ^15^N-methylpyridinium. ULF NMR spectra of ^15^N-methylpyridinium (^15^N-MP) in **a** H_2_O and **b** D_2_O measured after different storage times. The peak at ≈ 80 Hz corresponds to the Larmor precession frequency of water protons while the satellite peaks correspond to the Larmor precession of ^15^N-MP protons (coupled to ^15^N). **c** Decay of the proton signals shown in **a**, ^15^N-MP plus H_2_O peaks (green triangles) and the proton signals shown in **b**, the ^15^N-MP peaks in D_2_O (red circles), as a function of storage time. The solid lines are the fit overlaying the data points. For the case of ^15^N-MP dissolved in D_2_O, the line is a monoexponential decay with *T*_1_ time of 2.38(3) s. For the case of ^15^N-MP dissolved in H_2_O, the line is a bi-exponential decay with *T*_1_ times of 4.8(5) s and 1.24(3), for ^15^N-MP and water protons, respectively.

Figure 3a shows the spectrum of ^15^N-MP dissolved in H_2_O. The spectrum consists of a strong peak at about 80 Hz, arising from water protons, and two broader satellite humps, symmetric around the proton peak (75-85 Hz). The latter are groups of overlapping peaks, originating from the precession of ^15^N-MP protons coupled to ^15^N. When the storage time (in a storage field of 10 μT) was increased, a water-proton peak decayed faster than the ^15^N-MP peaks (Fig. 3a). This reveals a shorter longitudinal relaxation time of the water protons than for the ^15^N-MP protons. To calculate the *T*_1_ lifetimes of the protons, data points, corresponding to the normalized integration values of proton peaks in the 75-85 Hz frequency range, were plotted as a function of the storage time (Fig. 3c). Due to the overlap between the water- and MP-proton peaks, the data (denoted by green triangles) was fit to bi-exponential decay. This allowed us to determine the ^15^N-MP and water-proton relaxation times as 4.8(5) and 1.24(3) s, respectively. A similar study was performed for ^15^N-MP dissolved in D_2_O, where only the ^15^N-MP peaks were observed. Figure 3c shows the amplitude of the peak measured at 81 Hz versus the storage time. This data was fit with a single exponential decay and the longitudinal relaxation time *T*_1_ of 2.38(3) s was determined. These results reveal an interesting effect of the longer relaxation time of ^15^N-MP protons in water compared to that in the deuterated solution. A similar solvent effect on the *T*_1_ relaxation of both ^15^N and ^1^H in ^15^N-MP was also observed in high-field NMR (see Supplementary Table 1 and Supplementary Fig. 1 in Supplementary Note 1). This result might be explained by the isotope effect in which the solvents differ in their hydrogen bonding characteristics and the formation of the solvation spheres^46,47^. In addition, lower mobility of D in D_2_O compared to H in H_2_O may result in slower dipole reorientation, hence a higher relaxation rate is expected in D_2_O, especially of small ionic compounds^48^. Despite these speculations, better understanding of relaxation mechanisms of coupled spin systems at ZULF conditions needs further studies.

One should point out that low-frequency peaks of ^15^N nuclei that are coupled to the protons were not visible in the ULF spectra of ^15^N-MP. This is not surprising because, due to low gyromagnetic ratio of ^15^N nuclei, the signal from coupled ^15^N-spin precession in the ULF regime is expected to be approximately two orders of magnitude smaller than the signals from protons. To remedy that, parahydrogen-based hyperpolarization techniques can be employed to study ^15^N nuclei under the ZULF regime even using unlabeled compounds (natural abundance of ^15^N = 0.36%) as it was recently demonstrated^49,50^.

### Paramagnetic relaxation in ULF NMR

**Fig. 4:**
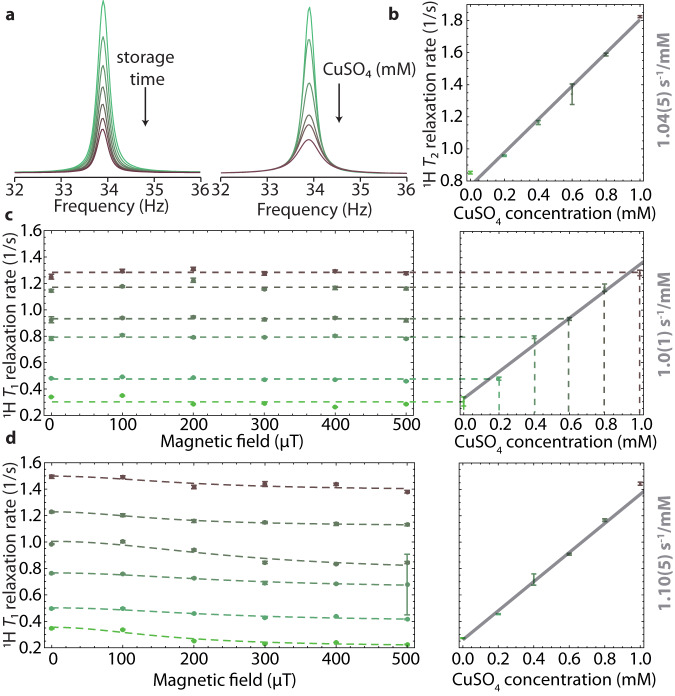
Relaxation dispersion profile of aqueous CuSO_4_ solutions in ultra-low field. **a** ULF NMR proton spectra of aqueous solutions of CuSO_4_ measured in a field of 0.8 μT. The water-proton peak is shown as a function of the storage time (without CuSO_4_ added) and as a function of CuSO_4_ concentration; the storage field was 10 μT. **b** *T*_2_ relaxation rate as a function of the CuSO_4_ concentration (obtained from the average of vacuumed and non-vacuumed samples) and the linear fit with the extracted value of relaxivity. **c** Longitudinal relaxation rate 1/*T*_1_ of the water proton in CuSO_4_ solutions without and **d** with degassing prior to the measurements as a function of the magnetic field (left panel) and CuSO_4_ concentration (right panel). The dashed line in the field profile corresponds to the fit of Lorentzian function ${T}_{1}^{-1}(B)={T}_{1}^{-1}(0)/(1+{(\gamma B)}^{2}{\tau }_{c}^{2})$^14^, with the correlation time *τ*_*c*_ set to 18 μs, providing an adequate match to observed experimental results. The relaxivity values are extracted from the linear fit to the average relaxation rates for various concentrations of CuSO_4_ across different magnetic fields. Error bars indicate standard-deviation errors of the fitting parameters.

To demonstrate the feasibility of ULF NMR relaxometry for the analysis of biological samples, aqueous solution of paramagnetic copper sulfate, CuSO_4_, widely used for quantitative imaging studies, was investigated. This compound was used to prepare phantoms that mimic biological samples with short relaxation times^32^. Water-proton precession was observed to gather information about the *T*_1_ and *T*_2_ proton relaxation, which is accelerated by the presence of paramagnetic ions in the solution. During the ULF spectra acquisition, the NMR sample was subjected to a detection field of 0.8 μT, corresponding to proton Larmor frequency of about 34 Hz. In Fig. 4a, ULF NMR spectra corresponding to water-proton precession, are shown for different storage times in a field of 10 μT. The effect of CuSO_4_ is observed as a broadening of the ULF NMR peak (Fig. 4a). The longitudinal (1/*T*_1_) and transverse (1/*T*_2_) relaxation rates, measured for water in different CuSO_4_ concentrations, are shown in Fig. 4b–d. The data demonstrate that the presence of up to 1 mM of paramagnetic CuSO_4_ accelerated the longitudinal and transverse relaxation by an order of magnitude. According to the general relaxation theory^51^, the 1/*T*_1_ and 1/*T*_2_ relaxation rates are proportional to the concentration of the paramagnetic species and can be calculated by adding the paramagnetic and diamagnetic relaxation rates. Following this approach, *T*_2_ relaxivity of CuSO_4_ is calculated as 1.04(5) s^−1^/mM (Fig. 4b). *T*_1_ relaxivity of CuSO_4_ is studied for magnetic fields between 10 μT and 510 μT, which allowed us to reconstruct the NMRD profiles (Fig. 4c, d).

The longitudinal relaxation rates 1/*T*_1_, derived from ULF NMR measurements with non-degassed CuSO_4_ water solutions (flame-sealed without the freeze-pump-thaw procedure), are presented in Fig. 4c. Overall, no significant dependence of the longitudinal relaxation rate on the magnetic-field strength was observed. The extracted *T*_1_ relaxivity of CuSO_4_ was 1.0(1) s^−1^/mM. On the other hand, measurements with the non-degassed pure water solution (bottom dashed line in Fig. 4c) and degassed CuSO_4_ water solutions (Fig. 4d) show that the gradual increase of the storage field from 10 μT to 510 μT resulted in a continuous decrease in the longitudinal relaxation rate 1/*T*_1_. The possible explanation for this difference can be the simultaneous presence of paramagnetic oxygen and CuSO_4_ in solutions. This may accelerate the relaxation process resulting in the disappearance of the weak dependence of water-proton relaxation on the storage field in the ULF regime. It has also been shown that the dispersion curve obtained from CuSO_4_ solutions is rather flat compared to those from most biological tissues^52^. *T*_1_ relaxivity of CuSO_4_ is calculated as 1.10(5) s^−1^/mM using 1/*T*_1_ values averaged over different magnetic fields. In both cases, *T*_1_ relaxivity constant of CuSO_4_ in water are in good agreement with literature values obtained in high magnetic field^53^.

To test the detection limit of ULF NMR relaxometry, even higher concentrations of CuSO_4_ were studied. In water samples containing 1.5 and 2 mM of CuSO_4_, stored in a field of 10 μT, the longitudinal relaxation times *T*_1_ of protons were 0.38(5) and 0.363(7) s, respectively. It should be stressed that, despite the fast relaxation, the water-proton peak was clearly visible in the ULF NMR spectrum, allowing relaxation studies. Since the proton relaxation time of the blood is short as well, i.e., on the order of hundreds of milliseconds (see below), this phantom study has shown the feasibility of ULF NMR relaxometry of blood using an atomic magnetometer^3^.

### ULF relaxometry of whole blood and plasma

In this part of the study, we used human whole-blood and blood-plasma to demonstrate the applicability of ULF NMR relaxometry for biological samples. Blood with all its components intact (white and red blood cells, platelets, and plasma, collected using a tube that contains an anticoagulant solution) is called whole blood. Blood plasma (45% of the whole blood) is the cell-free supernatant, which can be obtained by centrifuging whole blood. The high water content of whole blood and plasma allowed us to readily observe proton signals under ULF conditions.

**Fig. 5:**
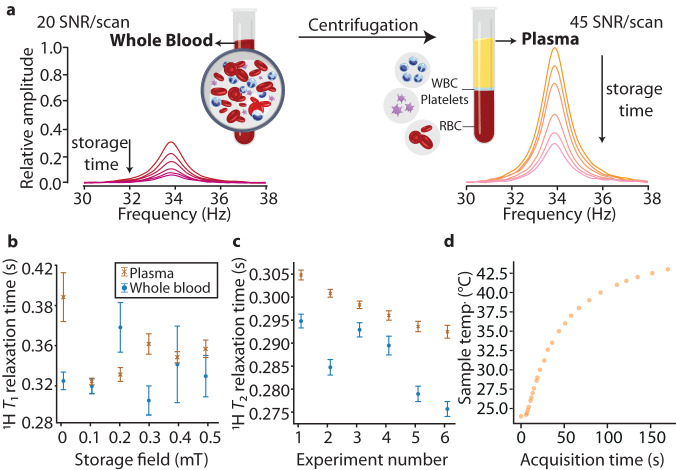
Relaxation of human whole-blood and blood-plasma in ultra-low field. **a** Proton ULF NMR signals from human whole-blood (left) and blood-plasma (right) samples shown as a function of storage time at the 10 μT field. Transverse magnetic field during the detection was 0.8 μT. Signal-to-noise ratio (SNR) per scan is indicated for each sample. **b** *T*_1_ and **c** *T*_2_ relaxation times of human whole-blood and blood-plasma at ULFs (taken at the storage fields between 10 and 510 μT). It was found that the *T*_2_ relaxation times (extracted from the linewidth) decreased with increasing the number of experiments performed on the same sample at the same detection field. Error bars indicate standard-deviation errors of the fitting parameters. **d** Temperature of the NMR sample after being placed near the two commercial magnetometers. In a typical ZULF NMR experiment, a sample spends an equal amount of time in a prepolarization magnet (at ≈ 22^ ∘^C) and next to the two magnetometers, which leads to the considerable heating of the liquid over long experiments.

Figure 5 shows that the amplitude of the proton signal was larger in plasma samples than in whole-blood samples. This is an effect of higher water content and slower proton relaxation in plasma compared to the whole blood, where the cellular component is dominated by hemoglobin-rich red blood cells^54^. At 10 μT, proton relaxation times were determined as: ${T}_{1}^{{{{{{\mathrm{blood}}}}}}}=0.305(9)$ s, ${T}_{2}^{{{{{{\mathrm{blood}}}}}}}=0.295(2)\,{{{{{{{\rm{s}}}}}}}}$; ${T}_{1}^{{{{{{\mathrm{plasma}}}}}}}=0.39(3)\,{{{{{{{\rm{s}}}}}}}}$, ${T}_{2}^{{{{{{\mathrm{plasma}}}}}}}=0.304(1)\,{{{{{{{\rm{s}}}}}}}}$. As shown, the difference in *T*_1_ between whole blood and plasma is higher than in *T*_2_, which reveals the stronger influence of the cell components on the *T*_1_ relaxation and agrees with low-field NMR relaxometry^3^.

Especially in venous whole-blood samples, intracellular heterogeneous distribution of the paramagnetic molecules, such as deoxygenated hemoglobin, might cause magnetic-susceptibility difference between the blood cell and plasma, which contributes to the *T*_2_ relaxation by dephasing water-proton coherence^55^. However, this mechanism becomes insignificant in ULF regime, which allows one to measure heterogeneous samples without susceptibility broadening^56^.

To determine the limitations of the method, the ULF relaxation profile of human whole-blood and blood-plasma was measured. As shown in Fig. 5b, c, the *T*_1_ and *T*_2_ relaxation times of the protons in whole blood varied with the storage field (10–510 μT) without a specific pattern. This can be a result of a residual cellular metabolism, which can change the protein content over time^57^. In blood plasma, where no cellular metabolism is expected, the content of metabolites should remain stable over time. In contrast to whole blood, in plasma, the decrease in the *T*_1_ relaxation time of protons was observed with the variation of the storage field from 10 μT to 510 μT (Fig. 5b). This complicated dependence of the relaxation times on the storage field can be attributed to a sample heating in the proximity of an operating atomic magnetometer. Indeed, in our magnetic-field profile measurements, the storage field was increased step by step without changing the sample. Even though the *T*_2_ relaxation time (extracted from the linewidth) was measured at the same detection field, we observed its decrease in the subsequent experiments (Fig. 5c). This additionally confirms the effect of temperature on the measured relaxation times. When the sample temperature was measured by thermocouple as a function of acquisition time (time the sample spent near the atomic magnetometers), we observed that it could exceed 40^∘^C, as shown in Fig. 5d. Therefore, the repeated measurements might cause an increase in the temperature of the sample over time and could cause faster relaxation^58^. To overcome this limitation, active cooling or/and temperature stabilization can be implemented in the setup to maintain temperature stability^14^. It should also be noted that the *T*_2_ values are consistently lower than the *T*_1_ values, which resulted from the additional contribution of low-frequency fluctuations such as slow molecular rotation, chemical exchange, and diffusion to the transverse relaxation^2^.

## Conclusions

In this work, ZULF NMR relaxometry was exploited to collect information about the molecular environment in chemical solutions and biofluids. The strong influence of paramagnetic oxygen on zero-field NMR spectroscopy was demonstrated by measuring spin systems with ^1^H-^31^P and ^1^H-^13^C couplings. In particular, we showed a strong dependence of the heteronuclear singlet-state lifetime on the concentration of dissolved oxygen. We also investigated the lifetime of ^15^N-methylpyridinium, a precursor of potential contrast agents, in the ULF regime. In particular, we demonstrated slower longitudinal relaxation of this compound in H_2_O compared to D_2_O. Nuclear magnetic relaxation dispersion profiles of aqueous solutions of a paramagnetic compound (CuSO_4_) were studied in the ULF regime. We demonstrated that samples with and without dissolved oxygen exhibit a slightly different magnetic-field dependence, which agrees well with the relaxation theory, proving the reliability and potential applicability of the method for chemical analysis. Finally, we investigated the ULF NMR spectra of human whole-blood and blood-plasma. Despite the shorter (≈300 ms) relaxation times of these biofluids, the high-quality ULF NMR spectra were obtained and examined. These measurements hold promise as a new blood diagnostic technique and a comprehensive, systematic study on biological fluids is a subject of ongoing research. Without the need for sophisticated sample preparation, the blood *T*_2_ relaxation time obtained in short measurements (<1 min) at the ULF conditions is a potentially valuable marker of inflammation often induced by metabolic disorders, infections, etc. Our work points towards the applicability of ZULF NMR relaxometry employing atomic magnetometers as a simple, portable, robust, inexpensive, and sensitive tool in chemical and biological analysis. In addition, ULF NMR relaxometry of biofluids paves the way for molecular imaging at ZULF conditions where *T*_1_ and *T*_2_ relaxation properties of specific molecules predominately determine image contrast^59–62^.

## Methods

### Sample preparation

Trimethyl phosphate (CAS# 512-56-1) and ^13^C-formic acid (CAS# 1633-56-3) were purchased from Sigma-Aldrich. For each compound, four 0.2 ml neat liquid samples were prepared by transferring them into standard 5 mm NMR tubes. To investigate the effect of oxygen on relaxation times in each compound, one sample was sealed without being degassed. The other three samples were degassed once, twice, and three times using the freeze-pump-thaw method with the use of a two-stage vacuum pump. This allowed us to evacuate the gas above a sample to a gas-pressure of 10^−6^ mbar. After degassing, the tubes were flame-sealed under vacuum.

^15^N-methylpyridinium was synthesized according to literature procedures starting from ^15^N-enriched pyridine^63^ and dissolved in D_2_O and H_2_O by obtaining 5.5 M ^15^N-methylpyridinium solutions. The solutions were transferred to 5 mm NMR tubes and then tubes were flame-sealed under vacuum following two freeze-pump-thaw cycles for degassing. For high-field relaxometry studies, ^15^N-methylpyridinium was dissolved in D_2_O and 1:4 D_2_O and H_2_O mixture at 5.5 M concentration. The samples were degassed prior to measurements.

Copper sulfate (CuSO_4_) (CAS# 7758-98-7) was also purchased from Sigma-Aldrich. The CuSO_4_ solutions with various concentrations were prepared by dissolving the compound in deionized water. For each concentration, 0.2 ml of solutions were placed inside two standard 5 mm NMR tubes. One of the tubes was sealed without degassing, and another tube was degassed twice using the freeze-pump-thaw method and then flame sealed. The pressure above the frozen liquid after the second cycle was below 10^−4^ mbar.

Human whole-blood samples from healthy adults (male volunteers) were collected in tubes containing heparin as an anticoagulant (volume ratio: 9:1) on the day of the experiment (blood came from the Regional Blood Transfusion Centre, Kraków, Poland). The volunteer donors had not taken any medicines for the two weeks prior to the withdrawal. Informed consent was given by each volunteer prior to the blood withdrawal and the study was in accordance with the principles outlined in the World Medical Association (WMA) Declaration of Helsinki, as well as Bioethical Commission of the Jagiellonian University. 300 μL of whole blood were kept for the measurements. Remaining whole blood was subjected to centrifugation (acceleration of 800 × *g* and run time: 15 min at room temperature, with soft stop) to separate plasma from the rest of the blood components. Plasma was collected and placed in Eppendorf tubes for further sample preparation procedures and measurements. 0.2 ml of whole blood and plasma were transferred to NMR tubes and flame-sealed without degassing. The samples were kept at 4^∘^C to reduce the metabolism until measurement and measured up to an hour after blood withdrawal.

### Experimental setup

ZULF NMR experimental setup was built/designed by DM Technologies, Liszki, Poland (dmtechnologies.eu). For the relaxation study using NMR at zero magnetic field, the NMR samples, prepolarized for about 20 s inside a permanent magnet (1.4 T), were mechanically shuttled to the zero-field region (interior of a multi-layer magnetic shield) through a guiding field (10 μT) applied by the solenoid (Fig. S1). After transfer to the detection region, the samples were stored in the guiding field of the solenoid (storage field) for variable times (from 1 to 15 s). After that time, the storage field was switched off and an optimal transverse pulse was applied to generate a zero-field NMR signal of maximum amplitude^34^. For the ^1^H-^13^C spin system, the optimal pulse-induced rotation angle was 4*π* for the ^1^H spins, while in the ^1^H-^31^P system, the maximum amplitude was achieved using 5*π* pulse angle for the ^1^H spins.

To investigate the relaxation of water protons in CuSO_4_ solutions, whole blood, plasma, and ^15^N-coupled-proton relaxation in ^15^N-methylpyridinium solutions, ULF NMR spectroscopy was used (Fig. S1). In these cases, after 15 s thermal prepolarization with the 1.4 T permanent magnet, the samples were transferred to the detection region (inside the magnetic shield), where it was stored in various guiding fields (10–510 μT) and times (0.5–6.05 s). Then the guiding field was switched off (within 10 μs) while a detection field of 0.8 μT, corresponding to about 34 Hz of precession of ^1^H, was applied in a transverse direction to the sensitive axis of the magnetometer for water-proton relaxation studies. In the ^15^N-coupled-proton relaxation measurements, the detection field was adjusted to 1.88 μT (corresponding to 80 Hz of ^1^H precession). The detection fields were chosen in such a way that the proton-precession signal appeared in a convenient spectral region, i.e., far from 50 Hz magnetic noise and its overtones but also away from low-frequency flicker noise. It should be noted that a further increase in the solenoid storage field could introduce field inhomogeneities, which may affect the *T*_2_ relaxation. For detailed schematics of our ZULF NMR experimental setup and the experimental sequence see Supplementary Note 2 and Supplementary Fig. 2. The characterization of magnetic field homogeneity for the detection field is presented in Supplementary Note 3 and Supplementary Fig. 3.

High-field *T*_1_ measurement on ^15^N-methylpyridinium samples was performed via an inversion recovery experiment on a 7 T Bruker NMR system with Avance III console.

### Data analysis

Each ZULF NMR measurement consisted of 4–64 repetitions. In the *T*_1_ studies, each data point was obtained after averaging transients. Relaxation times *T*_1_ were calculated by fitting the data with exponential decay. The transverse longitudinal relaxation times *T*_2_ were calculated from the linewidths of the peaks in the spectra. Full maximum half-width (FWHM) values were derived by fitting NMR spectra to the Lorentzian function. Standard-deviation errors of the fitting parameter are denoted with error bars. Data processing and data analysis were performed using custom Python scripts described in ref. ^34^. The statistical analysis was performed using the OriginPro software (version 2020; Origin Lab Corporation, USA). A two-sample *t*-test was used to compare the linewidth of zero-field NMR peaks after the different number of freeze-pump-thaw cycles.

### Supplementary information

Supplementary Information

## Data Availability

All raw NMR data is publicly available through the Dryad repository (https://doi:10.5061/dryad.nk98sf7z7).
