# Author Correction: Zero- to low-field relaxometry of chemical and biological fluids

**DOI:** 10.1038/s42004-023-00980-9

**Published:** 2023-08-17

**Authors:** Seyma Alcicek, Piotr Put, Adam Kubrak, Fatih Celal Alcicek, Danila Barskiy, Stefan Gloeggler, Jakub Dybas, Szymon Pustelny

**Affiliations:** 1 Goethe University Frankfurt, University Hospital, Institute of Neuroradiology, 60528 Frankfurt am Main, Germany; 2 Institute of Physics Faculty of Physics, Astronomy and Applied Computer Science, Jagiellonian University in Kraków, 30-348 Kraków, Poland; 3 Faculty of Chemistry, Jagiellonian University in Kraków, 30-387 Krakow, Poland; 4 Jagiellonian Center for Experimental Therapeutics, Jagiellonian University in Kraków, 30-348 Kraków, Poland; 5 Helmholtz Institute Mainz, GSI Helmholtz Center for Heavy Ion Research GmbH, 55128 Mainz, Germany; 6 Institute of Physics, Johannes Gutenberg-Universität, 55128 Mainz, Germany; 7 Max Planck Institute for Multidisciplinary Sciences, 37077 Göttingen, Germany

**Keywords:** Biophysical chemistry, Solution-state NMR, Sensors, Biophysical chemistry, Blood proteins

Correction to: *Communications Chemistry* 10.1038/s42004-023-00965-8, published online 04 August 2023.

The original version of this Article contained errors in the author affiliations.

Authors Piotr Put and Szymon Pustelny were incorrectly listed as being affiliated with Goethe University Frankfurt in lieu of Jagiellonian University.

This has now been corrected in both the PDF and HTML versions of the Article.

