# Supplementary material for: Zero- to low-field relaxometry of chemical and biological fluids
Source: Commun Chem. 2023 Aug 4;6:165. doi: 10.1038/s42004-023-00965-8 (PMC10403525; doi:10.1038/s42004-023-00965-8)
Supplement: Supplementary file 1 — Supplementary Information [file 42004_2023_965_MOESM1_ESM.pdf]

Supplementary Information: Zero- to Low-field Relaxometry of Chemical and Biological Fluids

Seyma Alcicek,[1,2,*] Piotr Put,[2] Adam Kubrak,[3] Fatih Celal Alcicek,[4] Danila Barskiy,[5,6] Stefan Gloeggler,[7] Jakub Dybas,[4] and Szymon Pustelny[2,†]

[1] *Goethe University Frankfurt, University Hospital, Institute of Neuroradiology, Frankfurt am Main, 60528, Germany*

[2] *Institute of Physics, Faculty of Physics, Astronomy and Applied Computer Science, Jagiellonian University in Kraków, 30-348 Kraków, Poland*

[3] *Faculty of Chemistry, Jagiellonian University in Kraków, 2 Gronostajowa St., 30–387, Krakow, Poland*

[4] *Jagiellonian Center for Experimental Therapeutics, Jagiellonian University in Kraków, 14 Bobrzynskiego St., 30–348, Kraków, Poland*

[5] *Helmholtz Institute Mainz, GSI Helmholtz Center for Heavy Ion Research GmbH, 55128 Mainz, Germany*

[6] *Institute of Physics, Johannes Gutenberg-Universität, 55128 Mainz, Germany*

[7] *Max Planck Institute for Biophysical Chemistry, Am Fassberg 11, 37077, Göttingen, Germany*

Supplementary Note 1: T_1 relaxation of ^{15}N-methylpyridinium at high magnetic field

To further investigate atypical relaxation properties of ^{15}N-methylpyridinium in H_2O and D_2O solutions (see main text), we performed additional high-field study of T_1 relaxation of this compound. The high-field, chemical-shift-resolved ^{1}H spectra of ^{15}N-methylypridium in D_2O and in the mixture of D_2O and H_2O are shown in Supplementary Figure 1. The data reveals a ≈ 0.6 ppm shift of the main peak towards smaller values in H_2O/D_2O solution. The high-field relaxation times T_1 of ^{1}H and ^{15}N, extracted from the experimental data, are shown in Supplementary Table 1. The results indicate 1.8-3.6 times longer longitudinal relaxation time T_1 when using non-deuterated water as a solvent. The increase in both ^{1}H and ^{15}N relaxation times is consistent with the ULF NMR data of strongly coupled heteronuclei presented in the main text.

* seyma.alcicek@kgu.de
† szymon.pustelny@uj.edu.pl

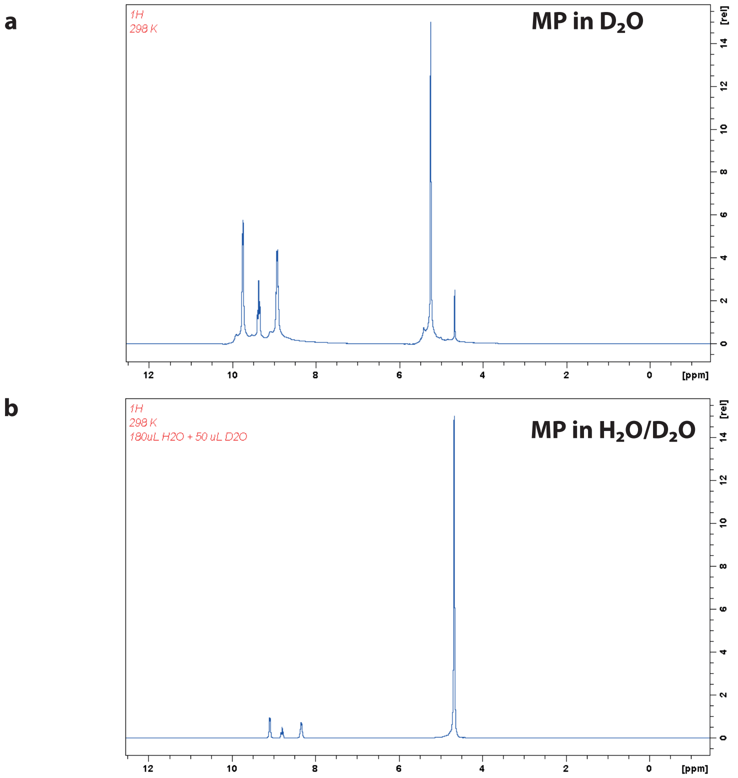

Supplementary Figure 1. ^{1}H spectra of ^{15}N-methylpyridinium in (a) D_2O and (b) H_2O/D_2O mixture in high field.

Supplementary Table 1. Relaxation time T_1 of ^{15}N-methylpyridinium measured at high (7 T) field.

^{1}H			
in D_2O		in H_2O/D_2O	
Chemical shift (ppm)	T_1 (s)	Chemical shift (ppm)	T_1 (s)
4.67	1.8	4.67	3.2
5.2	2.0	-	-
8.9	3.4	8.3	7.0
9.3	4.1	8.8	7.4
9.75	3.4	9.1	6.5
^{15}N			
in D_2O		in H_2O/D_2O	
201	40	201	144

Supplementary Note 2: Experimental setup and measurement sequence

Supplementary Figure 2 shows the experimental sequence (a) and experimental apparatus (b) used in ZULF NMR relaxometry. The traces illustrate the time profiles of magnetic fields in all spatial directions. After thermal prepolarization in the field of the permanent magnet of 1.4 T for the time τ_{pol} (≈ 20 s), the sample was mechanically shuttled to the ZULF detection region (inside a multi-layer magnetic shielding). The spatial orientation of sample polarization inside the shield was provided by the guiding field of a solenoid extending from the magnet to the detection region and eventually piercing the shield (not to generate magnetic field outside of the solenoid in the shield). After reaching the detection region, the sample was first stored for a given storage time τ_{pol} (up to 20 s) in a magnetic field ranging between 0 and 500 µT. Then, a guiding field was switched off (within 20 µs) and, if needed, a magnetic-field pulse of optimized amplitude B_{pulse} and length τ_{pulse} was used to generate ZULF NMR signals. The additional detection field B_{det} (from 0 to 0.1 µT) was applied during the ULF NMR measurements. Nuclear-spin evolution took place inside a four-layer magnetic shielding and it was measured with the use of an atomic magnetometer [1]. Shim coils,

placed inside the shield, were used to cancel residual magnetic fields and achieved ZULF conditions, while the pulse coils provided guiding fields and DC pulses to manipulate nuclear spins. For details of experimental apparatus and general ZULF NMR procedures see Ref. [2–5],

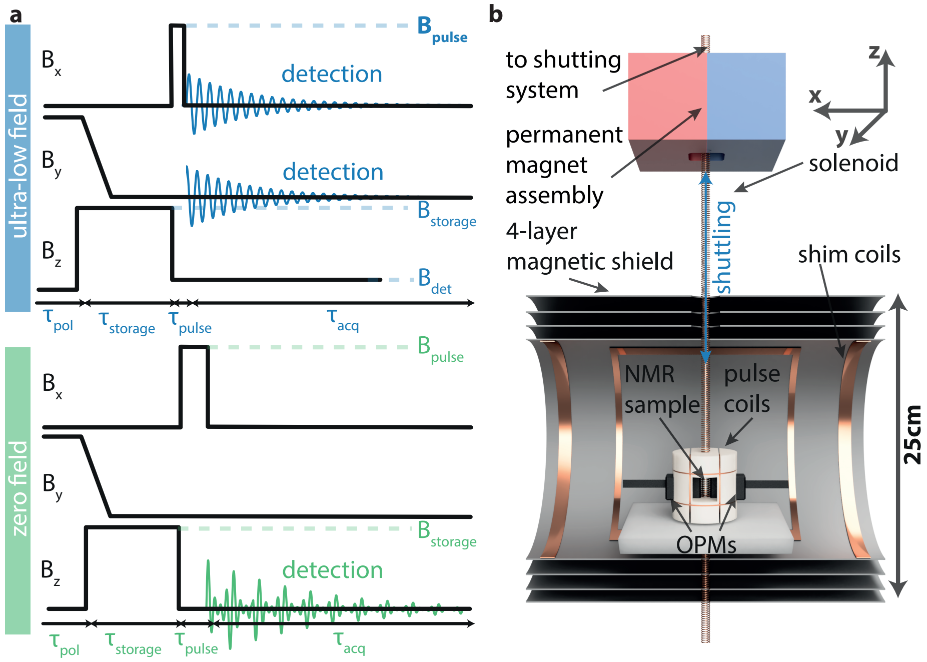

Supplementary Figure 2. (a) Measurement sequences (schematic magnetic field profiles) used in relaxometry studies performed under ultra-low field (top) and zero-field conditions (bottom). (b) Experimental setup used to perform ZULF NMR measurements, with a scale indicating real-world size of the NMR spectrometer.

Supplementary Note 3: Influence of magnetic field gradients on the extracted T_2 values

In the main text, the T_2 relaxation values extracted for a number of (bio-)chemical samples are determined directly from FID signal (i.e. from the width of the peak in the NMR spectrum). As this parameter is typically denoted in NMR literature as T_2^* and affected by the field gradients, the homogeneity of the detection field has been studied in detail and is presented in Supplementary Figure 3. To compare the T_2^* and T_2 in our experimental setup, T_2 of pure water was measured at an elevated magnetic field with the use of simple Hahn echo (Supplementary Figure 3b) experiment and compared with T_2^* extracted directly from NMR spectrum acquired at this elevated detection field (Supplementary Figure 3a). We can therefore estimate field gradient contribution to extracted T_2 taken at the detection field of 0.8 μT (34 Hz ^{1}H Larmor frequency) used in the ULF portion of this study:

$$1/T_{gradient} \approx (1/T_2^* - 1/T_2)\,\frac{34\,\mathrm{Hz}}{270\,\mathrm{Hz}} \approx \frac{1}{34\,\mathrm{s}}, \tag{S1}$$

which is less than 4% of relaxivity of pure water under the same field conditions, and therefore does not meaningfully affect T_2 times for methylpyridinium (Fig. 3 in the main text), $CuSO_4$ phantoms (Fig. 4 in the main text), blood and plasma (Fig. 5 in the main text) presented in the main text, all of which have shorter relaxation times than water. Please note that the residual inhomogeneity of the solenoid field does not effect studies taken in exactly zero magnetic field (Fig. 1 and 2 in the main text).

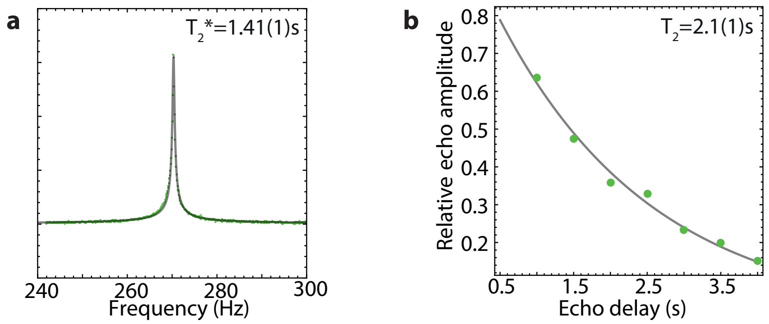

Supplementary Figure 3. Measurements characterizing homogeneity of the solenoid field. a) T_2^* measurement at elevated solenoid field and b) spin-echo measurement at the same magnetic field used to determine T_2.

Supplementary References

[1] D. Budker and D. Kimball Jackson, eds., *Optical Magnetometry* (Cambridge University Press, Cambridge, 2013).

[2] P. Put, *Ultra-Low and Truly Zero-Field Nuclear Magnetic Resonance*, Ph.D. thesis, Jagiellonian University, Krakow, Poland (2022).

[3] M. C. Tayler, T. Theis, T. F. Sjolander, J. W. Blanchard, A. Kentner, S. Pustelny, A. Pines, and D. Budker, Review of Scientific Instruments **88**, 091101 (2017).

[4] J. W. Blanchard, T. Wu, J. Eills, Y. Hu, and D. Budker, Journal of Magnetic Resonance **314**, 106723 (2020).

[5] P. Put, S. Pustelny, D. Budker, E. Druga, T. F. Sjolander, A. Pines, and D. A. Barskiy, Analytical Chemistry **93**, 3226 (2021).
